# Exercise performance and physiological responses: the potential role of redox imbalance

**DOI:** 10.14814/phy2.13225

**Published:** 2017-03-31

**Authors:** Kavey Vidal, Nathaniel Robinson, Stephen J. Ives

**Affiliations:** ^1^ Department of Health and Exercise SciencesSkidmore CollegeSaratoga SpringsNew York

**Keywords:** Antioxidants, exercise performance, fatigue, free radicals, ventilation

## Abstract

Increases in oxidative stress or decreases in antioxidant capacity, or redox imbalance, are known to alter physiological function and has been suggested to influence performance. To date, no study has sought to manipulate this balance in the same participants and observe the impact on physiological function and performance. Using a single‐blind, placebo‐controlled, and counterbalanced design, this study examined the effects of increasing free radicals, via hyperoxic exposure (F_i_O_2 _= 1.0), and/or increasing antioxidant capacity, through consuming an antioxidant cocktail (AOC; vitamin‐C, vitamin‐E, *α*‐lipoic acid), on 5‐kilometer (km) cycling time‐trial performance, and the physiological and fatigue responses in healthy college‐aged males. Hyperoxic exposure prior to the 5 km TT had no effect on performance, fatigue, or the physiological responses to exercise. The AOC significantly reduced average power output (222 ± 11 vs. 214 ± 12 W), increased 5 km time (516 ± 17 vs. 533 ± 18 sec), suppressed ventilation (*V*_E_; 116 ± 5 vs. 109 ± 13 L/min), despite similar oxygen consumption (*V*O_2_; 43.1 ± 0.8 vs. 44.9 ± 0.2 mL/kg per min), decreased *V*_E_/*V*O_2_ (35.9 ± 2.0 vs. 32.3 ± 1.5 L/min), reduced economy (*V*O_2_/W; 0.20 ± 0.01 vs. 0.22 ± 0.01), increased blood lactate (10 ± 0.7 vs. 11 ± 0.7 mmol), and perception of fatigue (RPE; 7.39 ± 0.4 vs. 7.60 ± 0.3) at the end of the TT, as compared to placebo (main effect, placebo vs. AOC, respectively). Our data demonstrate that prior to exercise, ingesting an AOC, but not exposure to hyperoxia, likely disrupts the delicate balance between pro‐ and antioxidant forces, which negatively impacts ventilation, blood lactate, economy, perception of fatigue, and performance (power output and 5 km time) in young healthy males. Thus, caution is warranted in athletes taking excess exogenous antioxidants.

## Introduction

Free radicals, such as reactive oxygen species (ROS), are highly reactive unstable atoms due to their unpaired number of electrons (Ji 1995). In vivo***,*** free radicals are typically produced either enzymatically (i.e., xanthine oxidase) or in the electron transport chain of the mitochondria, which can acutely impact cellular function, and ultimately damage membranes, cellular proteins, and DNA (Ji 1995; Lamina et al. 2013). Though, both enzymatic (e.g., superoxide dismutase) and non‐enzymatic circulating (e.g., tocopherols, ascorbate, etc.) antioxidants are able to cope with the production of free radicals, in health, under normal physiological conditions at rest, and during moderate exercise (Ji 1995; Lamina et al. 2013). Collectively, disturbance in the balance of free radicals and antioxidants, or redox balance, through excess free radical production, reduced antioxidant capacity, or both may have potent physiological impact.

Redox balance during exercise has been suggested to acutely influence skeletal muscle contractile capacity and ultimately the adaptation to exercise (Reid et al. 1994; Lamb and Westerblad 2011; Powers et al. 2011a,b; Westerblad and Allen 2011; Reid 2015; Cheng et al. 2016). Modest elevations in ROS within skeletal muscle, such as during mild to moderate exercise, have been suggested to improve, or even be required for, contractile function (Powers et al. 2011a; Cheng et al. 2016). In contrast, during high‐intensity, unaccustomed, or prolonged exercise, ROS accumulate and/or the antioxidant defense system may not be able to buffer the excessive exercise‐induced ROS, resulting in redox imbalance which has been shown to cause impair skeletal muscle contributing to peripheral fatigue (Reid et al. 1994; Reid 2015). Additionally, free radicals, specifically ROS, have also been documented to activate or sensitize afferent fibers (Houssiere et al. 2006; Delliaux et al. 2009), and thus could also conceivably contribute to altered physiological responses and/or perceptions of fatigue. Collectively, redox imbalance can contribute to peripheral, and possibly central, fatigue ultimately impairing exercise performance (Lamina et al. 2013). Thus, it has been suggested that increased antioxidant support through exogenous supplementation might inhibit fatigue and increase performance during high‐intensity exercise (Palazzetti et al. 2003, 2004).

Accordingly, antioxidant supplements such as vitamin C and vitamin E have been suggested to enhance exercise performance, however, the evidence is mixed (Jourkesh et al. 2007) or does not seem to support this claim (Kanter 1998; Zoppi et al. 2006; Reid 2008; Lamina et al. 2013). There is some evidence that suggests, thiol donors such as N‐acetylcysteine (NAC) might inhibit fatigue and improve performance (Reid et al. 1994; Medved et al. 2004; Reid 2008), but is only effective at clinical doses and is potentially rife with side effects and adverse reactions (Reid et al. 1994; Reid 2015). Thus, exploring other potential antioxidants is prudent. While results have been mixed regarding a positive benefit of antioxidants, such as Vitamin C (Kanter 1998; Lamina et al. 2013), questions remain whether a combination of antioxidants is required, what combination is optimal, and what the optimal dosing might be. To this end, an oral antioxidant cocktail (AOC), composed of vitamin C, vitamin E, and *α*‐lipoic acid, has been documented to significantly reduce free radicals (Richardson et al. 2007; Ives et al. 2014), suggesting that such a cocktail might mitigate excessive ROS without the myriad of potential side effects associated with NAC (Reid et al. 1994; Reid 2015). However, to date, no study has investigated whether this AOC is capable of improving exercise performance.

Additionally, while a line of research has sought to determine the potential benefit of antioxidants on reducing free radicals and performance (“antagonist approach”), no study has sought to experimentally induce free radicals in vivo in humans to determine potential impacts on performance (“agonist approach”). Previous research has shown that breathing supplemental oxygen, in those not medically indicated, increases reactive oxygen species and impairs redox balance (Marcus et al. 1992; Carpagnano et al. 2004; Ranadive et al. 2014), though it is unknown if such an alteration impairs performance. Indeed, correlative studies, such as those performed by Palazzetti et al. (2003, 2004) have demonstrated that high levels of endogenous free radicals produced during a 4‐week overtraining period were associated with impaired exercise performance. Though it has yet to be determined if acutely increasing free radicals, via exposure to hyperoxia, reduces exercise performance implicating ROS in fatigue of humans.

Therefore, the purpose of this study was to determine the impact of altering redox balance through consumption of an AOC (vitamin C, vitamin E, and *α*‐lipoic acid (Ives et al. 2014; Richardson et al. 2007)) and/or hyperoxic exposure (F_i_O_2_ = 1.0) on the physiological responses to exercise, performance, and fatigue in healthy active college‐aged males. Specifically, it was hypothesized that exposure to hyperoxia would reduce cycling exercise performance (5 km time, power output), disrupt physiological responses (ventilation, *V*O_2_, heart rate, blood lactate), and increase perception of fatigue (rating of perceived exertion, visual analog scale, handgrip maximal voluntary contraction), whereas the AOC would enhance exercise performance, enhance physiological responses, and decrease perception of fatigue. Lastly, if exposure to hyperoxia did alter performance, the physiological responses, or fatigue, we hypothesized the AOC would normalize the physiological responses, performance, and fatigue.

## Methods

### Participants

Fourteen healthy college‐aged (21 ± 0.3 years) males participated in the study. All participants were physically active (≥3 days/week), nonsmokers, and free of musculoskeletal problems and/or pain. Participants were screened using the health/fitness facility pre‐participation questionnaire from the American Heart Association/American College of Sports Medicine (AHA/ACSM). Those with cardiovascular, pulmonary, or metabolic disease or those taking regular medication (including antioxidant supplements) were excluded. All participants provided written informed consent prior to any testing. The protocol was approved by the Institutional Review Board of Skidmore College (IRB # 1412‐433) and is in accordance with the Declaration of Helsinki.

### Experimental design

**Figure 1:**
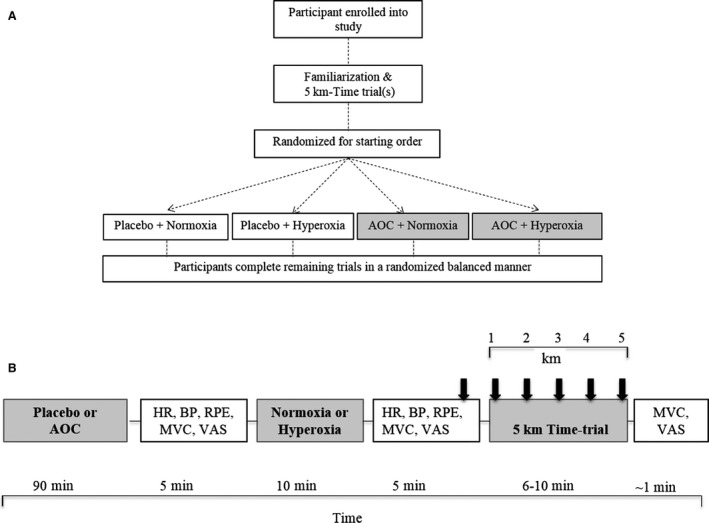
Overview of Study Design (Panel A). Overview of Experimental day (Panel B). Baseline measurements of heart rate (HR), blood pressure (BP), RPE, handgrip maximal voluntary contraction (MVC), and VAS were recorded before and after exposure to normoxia (F_i_O_2_ = 0.21) or hyperoxia (F_i_O_2_ = 1.0) for 10 min. | indicate blood draw for lactate before and at every kilometer during the 5 km time‐trial. HR, ventilation (*V*_E_), oxygen consumption (*V*O_2_), RPE, and RPM were recorded every minute during the 5 km time‐trial. Upon TT completion, MVC and VAS were recorded. RPE, ratings of perceived exertion; RPM, revolutions per minute; VAS, visual analog scale; BP, blood pressure; RPM, revolutions per minute.

The study used a balanced order, crossover design to investigate the potential impact of altering redox balance, via hyperoxic exposure, and antioxidant supplementation (antioxidant cocktail; AOC) on cycling performance, physiological responses, and perception of fatigue. All participants reported to the Skidmore College Human Performance Laboratory five times (within 2 weeks, >48 h apart), which included a familiarization and four experimental conditions. Prior to the experimental conditions, participants were familiarized with the respiration using a supported mouthpiece from a large reservoir balloon (120 L), the 5 km time‐trial on a cycle ergometer, and the measurement equipment. All tests were performed on a mechanically braked cycle ergometer (Monark 828E, Sweden) and participants were appropriately positioned with an upright posture and a 10‐degree knee bend at maximal leg extension, after which the settings were recorded for subsequent visits to ensure consistent positioning. Participants reported to the laboratory in a fasted state and without caffeine or alcohol use for 6 and 12 h, respectively. All participants were blinded to the four experimental conditions: (1) placebo + normoxia, (2) placebo + hyperoxia, (3) AOC + normoxia, and (4) AOC + hyperoxia. Participants the completed the four experimental conditions in a randomized, balanced, manner (for schematic view of experimental design, see Fig. 1).

### Antioxidant supplementation

All participants completed the study in a single blind, placebo controlled, counterbalanced design. Participants were asked to refrain from consuming excess antioxidant‐rich foods for at least 1 day before every visit, and to maintain a diet of similar composition throughout the duration of the study. Participants consumed the AOC or placebo in two doses separated by 30 min, with the first dose ingested 90 min before testing. The first antioxidant dose consisted of 300 mg of *α*‐lipoic acid, 500 mg of vitamin C and 200 I.U. (international units) of vitamin E taken 90 minutes before testing. The second antioxidant dose consisted of 300 mg of *α*‐lipoic acid, 500 mg of vitamin C and 400 I.U. of vitamin E taken 60 minutes before testing. This combination of antioxidants and dosing scheme used in the current study was specifically chosen since it has been previously documented to reduce plasma free radicals as detected by Electron Paramagnetic Resonance (EPR) Spectroscopy (Richardson et al. 2007) and has demonstrated efficacy without any side effects (Wray et al. 2009, 2012; Witman et al. 2012; Ives et al. 2014; Rossman et al. 2015). All antioxidants were obtained from United States Pharmacopoeia (USP) certified sources that adhere to Good Manufacturing Practice (GMP) standards. Placebo (PL) capsules, of similar appearance, were likewise consumed in two similarly timed doses. Participants were contacted and reminded to ensure the AOC would be taken at the right time prior to each study visit, and was confirmed upon arrival by bringing the empty bags in which the AOC or placebo were provided.

### Hyperoxic exposure

To experimentally‐induce an increase in free radicals, participants inspired air through a one‐way non‐rebreathe mouthpiece from a reservoir balloon (120 L), containing either normoxic room air (F_i_O_2_ = 0.21) or hyperoxic gas (F_i_O_2_ = 1.0) for a period of 10 min prior to exercise. Participants were blinded to which gas they were receiving. Breathing hyperoxic gas is an experimental approach that has been used and/or demonstrated to induce ROS or alterations in physiological function (Freeman et al. 1982; Mak et al. 2002; Houssiere et al. 2006; Gao et al. 2012; Ranadive et al. 2014; Caruana and Marshall 2015). Specifically, researchers (Mak et al. 2002; Gao et al. 2012) have found that a 10 min exposure is sufficient to induce physiological responses, which are reversible with Vitamin C, suggesting hyperoxia‐induced physiological changes are ROS dependent. As breathing hyperoxic gas during exercise is known to impact performance (Amann et al. 2006; Sperlich et al. 2016), we limited the exposure to hyperoxia to the pre‐exercise period only.

### Procedures

An overview of each experimental day is presented in Figure 1B. On arrival, height and body weight were recorded. After 5 min of quiet rest, baseline measurements of heart rate (HR; Polar, Lake Success, NY), systolic and diastolic blood pressure (SBP; DBP; American Diagnostic Corp.; Littman Quality) were taken. Capillary blood lactate (Lactate Pro, Australia) was also obtained via finger prick at rest prior to the 5 km time‐trial and at each kilometer during cycling performance. To assess fatigue, ratings of perceived exertion (RPE; Borg CR10 Scale) (Borg 1990) were obtained during the test, and a visual analog scale (VAS) for fatigue was assessed pre‐ and post‐exercise. Additionally, to determine potential effects of the AOC or hyperoxia exposure on central fatigue, we assessed handgrip maximal voluntary contraction (MVC_HG_), using a handgrip dynamometer (Takei, Japan) both before and after exercise. Three trials of MVC_HG_ were taken before inhaling the designated gas and the highest value was recorded. If we observed reductions in MVC_HG_, a muscle not directly involved in the cycling 5 km TT, this might be indicative of central fatigue and reduced global motoneuronal output (Amann et al. 2013).

Again, participants were blinded to condition (normoxia or hyperoxia), and treatment (placebo or AOC) they were assigned to at every visit. Baseline measurements were repeated after 10 min of mouthpiece breathing (F_i_O_2_ = 0.21 or 1.0). The participants were then positioned on the cycle ergometer, where the headgear and mouthpiece were placed onto the participant and connected to the metabolic cart (TrueOne 2400, Parvomedics, Sandy, UT). Participants were asked to cycle at a self‐selected resistance for 3 min as warm‐up. Thereafter, participants were instructed to perform the 5 km time‐trial as fast as possible, giving a maximal effort, at a resistance of 2.5 kp. In our laboratory repeatability of the 5 km TT is 3.3% coefficient of variation, intraclass correlation coefficient = 0.92 (*n* = 15, S. J. Ives, unpublished observations). Verbal encouragement was offered during each trial, in a consistent manner both within and between participants. During exercise participants!' ventilation rate (*V*_E_), oxygen consumption (*V*O_2_), HR, RPE, and revolutions per minute (RPM) were recorded at 1‐minute interval during the 5 km time‐trial. With the use of RPM recordings, power output (PO, in watts) was calculated as: PO = [(2.5kp × y RPM × 6 m) ÷ 6]. Cycling economy was calculated as *V*O_2_/power output (W). Blood lactate was measured every km until completion. Handgrip MVC and VAS for fatigue were measured immediately upon completion of the 5 km time‐trial. Recovery measurements of HR, BP and RPE were also taken post‐exercise.

### Statistical analysis

Statistical comparisons were performed with the use of the commercially available software (SPSS v. 22.0, IBM Inc., Armonk, NY). Paired *t*‐tests were used to identify potential changes in response to the breathing exposure, specifically normoxia versus hyperoxia. Repeated measures of analysis of variance (ANOVA) were used to determine if main effects were found in condition (normoxia vs. hyperoxia), treatment (placebo versus antioxidant cocktail), time points (minutes or kilometers), and the interaction between the three variables. Specifically, 2 × 2 (treatment × condition) repeated measures ANOVA were conducted for change in fatigue measures (MVC_HG_ and VAS), and multifactorial repeated measures of ANOVA (treatment × condition × time or distance) were conducted for performance and physiological measures. Tests of normality were performed, if a significant violation was found, the Greenhouse–Geisser correction was applied to the degrees of freedom. The level of significance was established at *P *<* *0.05. All data was expressed as means ± standard error (SE).

## Results

### Participant characteristics

Participant characteristics are presented in Table 1. All of the 14 healthy college‐aged participants completed the study and were compliant with the supplementation. At rest, there were no significant differences in response to either condition (normoxia vs. hyperoxia), treatment (placebo vs. AOC), or the interaction of condition and treatment on HR, MAP, sensation of fatigue, or muscle function (Table 2).

**Table 1:** Participant characteristics

	Mean ± SE
Age (year)	20.7 ± 0.3
Weight (kg)	77.2 ± 2.7
Height (cm)	179.3 ± 2.0
Body Mass Index (kg/m^2^)	24.0 ± 0.8

**Table 2:** Acute Effects of differing F_i_O_2_ (0.21 vs. 1.0) on HR, MAP, and Fatigue (VAS and HG MVC)

Treatment	Condition	HR (bpm)	MAP (mmHg)	VAS (0‐10 cm)	HG MVC (kg)
Placebo	Pre‐Normoxia	75 ± 3	83 ± 2	0.7 ± 0.3	50 ± 2
	Post‐Normoxia	74 ± 4	81 ± 2	0.7 ± 0.2	50 ± 2
Placebo	Pre‐Hyperoxia	73 ± 3	84 ± 3	0.9 ± 0.3	50 ± 2
	Post‐Hyperoxia	71 ± 3	81 ± 2	0.9 ± 0.3	49 ± 2
AOC	Pre‐Normoxia	74 ± 4	84 ± 2	0.8 ± 0.2	50 ± 2
	Post‐Normoxia	78 ± 4	83 ± 2	0.8 ± 0.3	49 ± 1
AOC	Pre‐Hyperoxia	76 ± 4	85 ± 2	1.1 ± 0.4	50 ± 2
	Post‐Hyperoxia	73 ± 4	85 ± 2	1.1 ± 0.4	49 ± 2
Means ± SE	*N* = 14				

AOC, antioxidant cocktail.

### Hyperoxic exposure, antioxidants, and exercise performance

**Figure 2:**
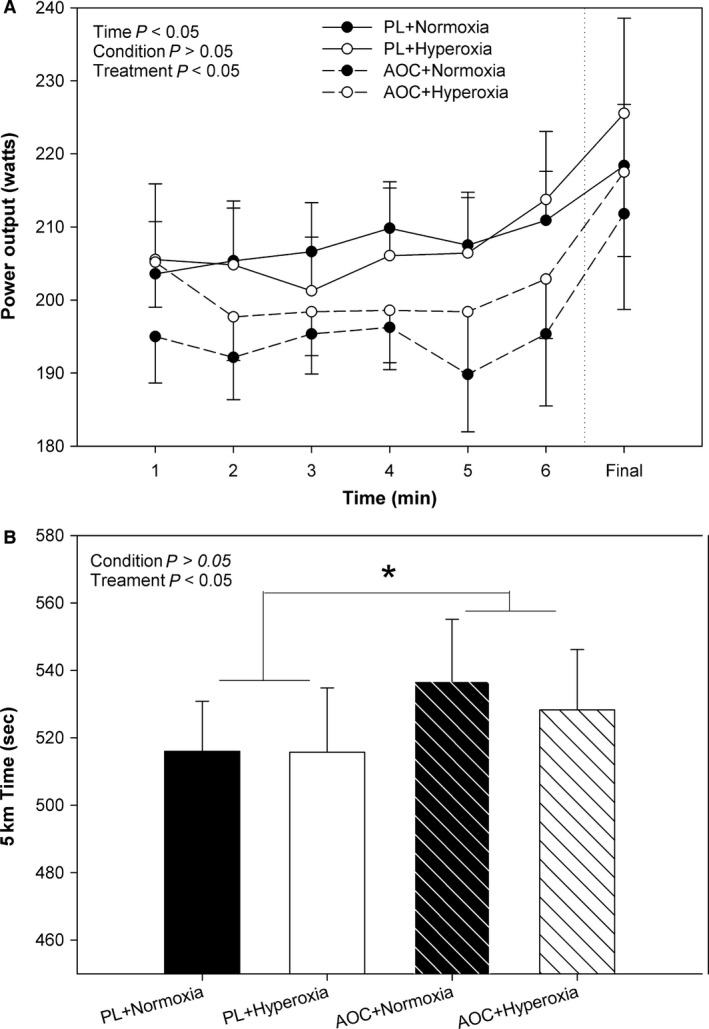
Effect of an Antioxidant Cocktail (AOC; treatment) and/or Hyperoxia (condition) on 5 km Time Trial (TT) Exercise Performance in Healthy College‐Aged Males (*n* = 14). (A) Power output during the 5 km TT, and (B) Time to complete the 5 km trial. PL; AOC. Values are means ± SE. *P < 0.05, main effect for treatment, PL vs. AOC. Means ± SE. PL, Placebo.

Power output varied significantly with time during the 5 km time‐trial (main effect for time, *P *<* *0.05), with an apparent sprint at the end of the TT (Fig. 2A). There was no significant main effect for condition (normoxia vs. hyperoxia) nor any significant interaction of condition and time or treatment and time (*P *>* *0.05) on power output. However, there was a main effect of treatment (placebo vs. AOC) on power output, where the AOC significantly reduced power output as compared to placebo (*P *<* *0.05, Fig. 2A).

For 5 km time, there was no significant main effect of condition (normoxia vs. hyperoxia) nor any interaction effects of condition and treatment (*P *>* *0.05, Fig. 2B). However, likely a result of reduced power output above, there was a significant main effect of treatment, specifically, the AOC resulted in a significantly (*P *<* *0.05) slower 5 km TT, by ~20 sec, as compared to placebo (Fig. 2B).

### Hyperoxic exposure, antioxidants, and physiological responses to exercise

**Figure 3:**
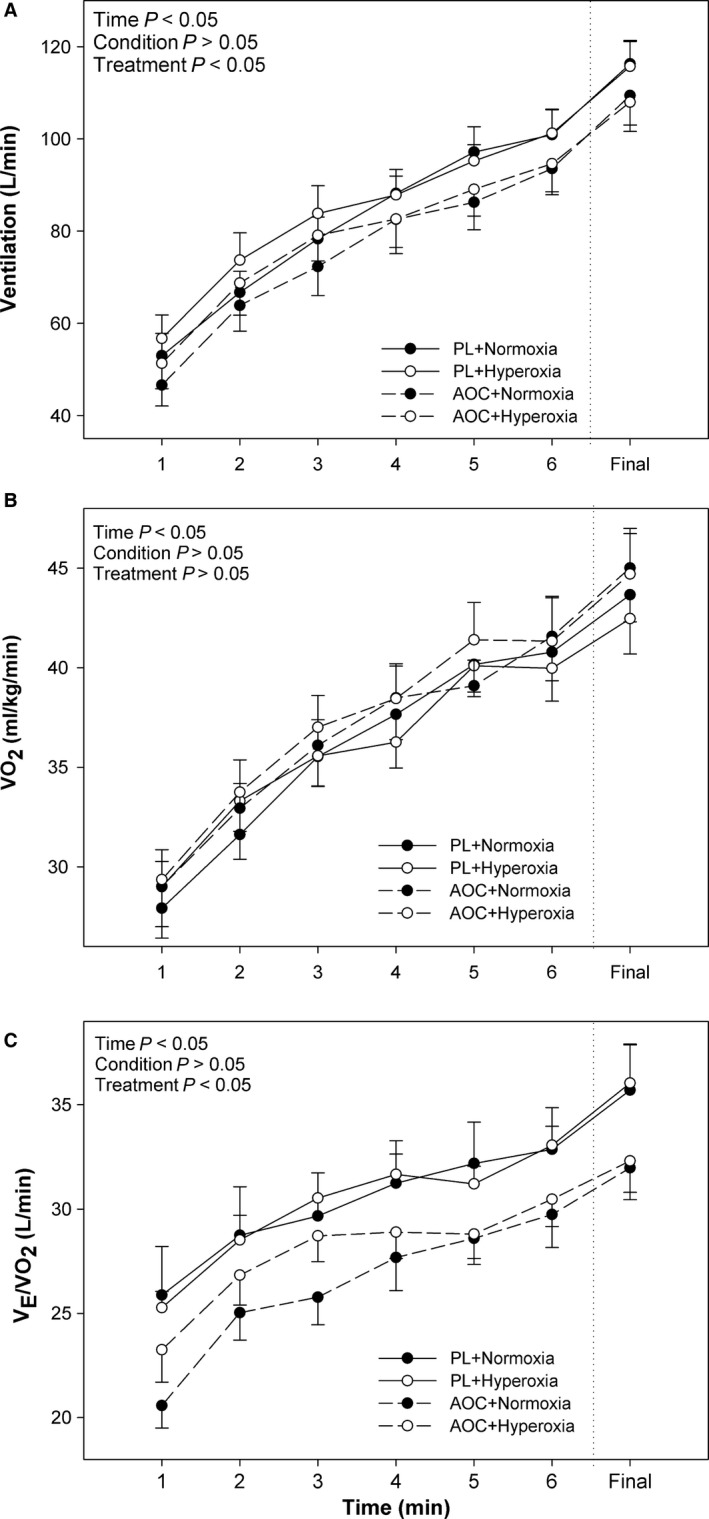
Effect of an Antioxidant Cocktail; treatment) and/or Hyperoxia (condition) on Pulmonary Responses during 5 km TT in Healthy College‐Aged Males (*n* = 14). (A) Ventilation, (B) Relative Oxygen Consumption (*V*O_2_), and (C) Ventilatory Equivalent for Oxygen (*V*_E_/*V*O_2_). To the right of the dashed vertical line, the last minute is shown, regardless of time to finish the 5 km. Data are means ± SE.

Ventilation (*V*_E_) increased significantly with time during the 5 km TT (*P *<* *0.05) (Fig. 3A). There was no significant effect (*P *>* *0.05) of condition (normoxia vs. hyperoxia), nor any interaction effects were observed for *V*_E_. However, there was a main effect for treatment in that the AOC significantly reduced *V*_E_ compared with the placebo (*P *<* *0.05, Fig. 3A). Oxygen consumption (*V*O_2_) also, increased significantly (*P *<* *0.05) over time during the 5 km TT (Fig. 3B). However, no significant effect of condition (normoxia vs. hyperoxia), treatment (placebo vs. AOC), or any interaction effects were observed (*P *>* *0.05) for *V*O_2_. The ventilatory equivalent for oxygen (*V*_E_/*V*O_2_) also increased significantly with time during the 5 km time‐trial (*P *<* *0.05, Fig. 3C). No significant effect of condition (normoxia vs. hyperoxia), or interaction effects were observed (*P *>* *0.05) for *V*_E_/*V*O_2_. However, there was a significant treatment (placebo vs. AOC) effect where the AOC did lower *V*_E_/*V*O_2_ versus placebo trials (Fig. 3C).

**Figure 4:**
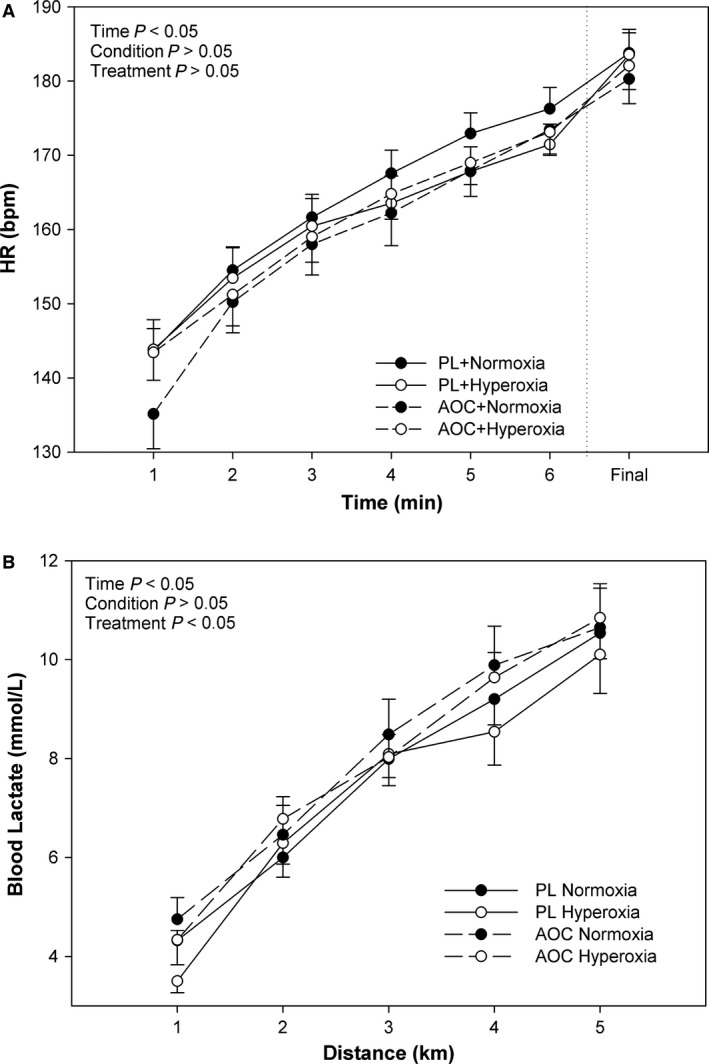
Effect of an Antioxidant Cocktail; treatment) and/or Hyperoxia (condition) on Heart Rate (HR, Panel (A) and Blood Lactate (Panel B) during 5 km TT in Healthy College‐Aged Males (*n* = 14). To the right of the dashed vertical line, the last minute of heart rate is shown, regardless of time to finish the 5 km trial. Data are means ± SE.

Heart rate increased significantly with time during the 5 km time‐trial (*P *<* *0.05, Fig. 4A). However, no significant effect of condition (normoxia vs. hyperoxia), treatment (placebo vs. AOC), or interaction effects were observed (*P *>* *0.05) for HR. Similarly, blood lactate levels increased significantly with time during the 5 km time‐trial (*P *<* *0.05, Fig. 4B). Although, no significant effect of condition (normoxia vs. hyperoxia) or interactions were observed (*P *>* *0.05), there was, however, a significant effect of treatment (AOC vs. Placebo) where higher blood lactate levels were observed with the AOC as opposed to placebo (*P *<* *0.05, Fig. 4B).

**Figure 5:**
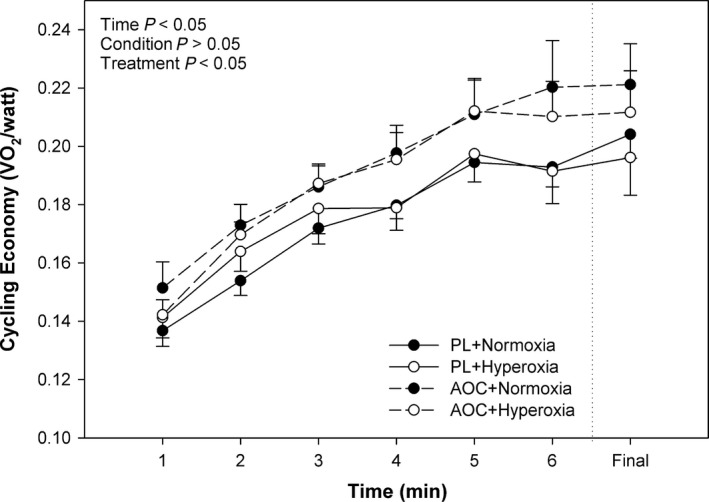
Effect of an Antioxidant Cocktail treatment and/or Hyperoxia (condition) on Cycling Economy during a 5 km TT in Healthy College‐Aged Males (*n* = 14). To the right of the dashed vertical line, the last minute of economy is shown, regardless of time to finish the 5 km trial. Data are means ± SE.

Economy (*V*O_2_/W) varied significantly (*P *<* *0.05) over time during the 5 km time‐trial (Fig. 5). There was no significant effect for condition (normoxia vs. hyperoxia), nor any interaction effects (*P *>* *0.05) for economy. Although, in addition to the decrements in performance above, there was a significant treatment effect where the AOC increased the *V*O_2_/W and reduced economy as compared with the placebo (*P *<* *0.05, Fig. 5).

### Hyperoxic exposure, antioxidants, and fatigue

**Figure 6:**
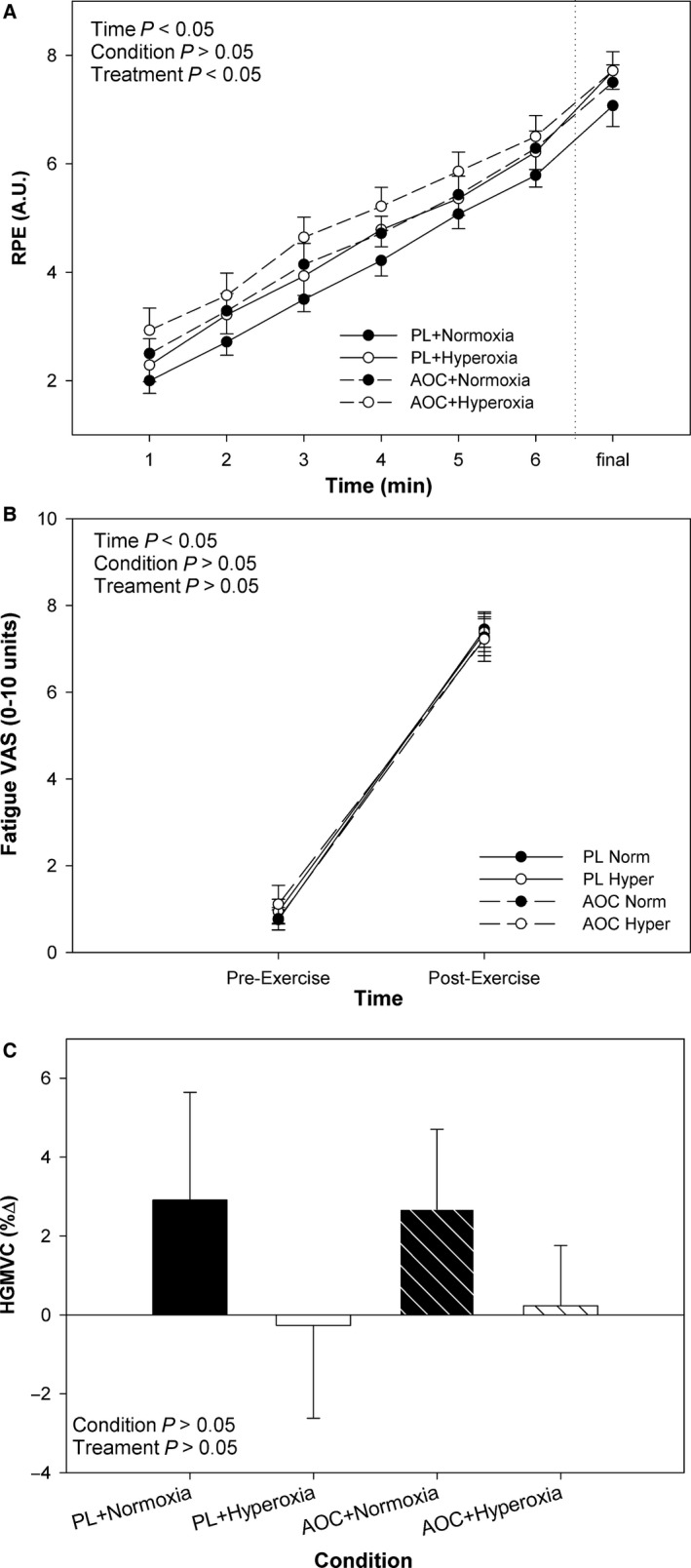
Effect of an Antioxidant Cocktail treatment and/or Hyperoxia (condition) on Fatigue in Response to a 5 km TT in Healthy College‐Aged Males (*n* = 14). To the right of the dashed vertical line, the last minute of economy efficiency is shown, regardless of time to finish the 5 km trial. Data are means ± SE.

Rating of perceived exertion (RPE) increased significantly with time during the 5 km time‐trial (*P *<* *0.05, Fig. 6A). There were no significant interaction effects (*P *>* *0.05) though there was a tendency for a main effect of condition (normoxia vs. hyperoxia) on RPE, where the hyperoxia trials tended to report higher RPE values, but did this not achieve statistical significance (*P *=* *0.09). There was a significant effect of treatment, where in the AOC trials, participants reported significantly higher RPE compared with the placebo (*P *<* *0.05, Fig. 6A).

VAS for fatigue increased significantly in response to the 5 km time‐trial (*P *<* *0.05, Fig. 6B). However, there was no significant effect of condition (normoxia vs. hyperoxia), treatment (placebo vs. AOC), or interaction effects (*P *>* *0.05) on fatigue VAS. Handgrip maximal voluntary contraction did not change significantly in response to the 5 km TT (*P *>* *0.05). Additionally, there was no significant effect of condition (normoxia vs. hyperoxia), treatment (placebo vs. AOC), or interactions (*P *>* *0.05) for MVC_HG_. However, a tendency was observed that the normoxic condition, independent of treatment, elicited ~3% increase in MVC_HG_ from pre‐ to post 5 km time‐trial, whereas hyperoxia elicited no visible change (Fig. 6C), but this was not statistically significant (*P *=* *0.08).

## Discussion

This study sought to determine if acutely altering redox balance, via ingestion of an antioxidant cocktail (AOC) and/or exposure to hyperoxia, would impact exercise performance, physiological responses, and perception of fatigue in healthy college‐aged males. In contrast to our hypotheses, hyperoxic exposure prior to the 5 km time‐trial had no effect on performance, physiological responses, or perception of fatigue. Moreover, the AOC significantly reduced exercise performance (5 km time, power output) as compared to the placebo. The AOC also altered the physiological responses, suppressing ventilation, *V*_E_/*V*O_2_, increasing lactate and the rating of perceived exertion, while maintaining a similar post‐exercise fatigue, despite a lower power output. Taken together, the results from the current study suggest that young healthy males are capable of handling increased free radicals associated with acute exposure to hyperoxia with no detriment to performance. In contrast, supplementing with antioxidants likely results in reductive stress, suggesting that an adequate level of free radicals are needed for proper physiological functioning and performance. Thus, in the rested healthy state, supplementation with antioxidants is unnecessary and are detrimental for physiology, performance, and fatigue.

### Antioxidants, hyperoxic exposure, and exercise performance

In the current study, contrary to our hypotheses, acute treatment with an AOC, of documented efficacy (Richardson et al. 2007), reduced exercise performance. It was also proposed that acute exposure to hyperoxia would have assumedly increased free radicals (Freeman et al. 1982; Mak et al. 2002; Carpagnano et al. 2004; Houssiere et al. 2006; Gao et al. 2012; Ranadive et al. 2014; Caruana and Marshall 2015), on top of any produced as a result of intense exercise (Bailey et al. 2003, 2004), reducing performance and enhancing fatigue. Finally, it was proposed that the AOC would restore performance and normalize perceptions of fatigue after the hyperoxic exposure. However, the hyperoxic exposure used in the current study had no effect on performance (Fig. 2). Additionally, irrespective of condition (normoxia vs. hyperoxia), the AOC likely induced reductive stress (Reid 2001; Powers et al. 2011a) thereby impairing performance.

Reid et al. (1993) proposed that modest amounts of reactive oxygen species (ROS) are actually required for optimal force production in skeletal muscle. However, deviation from this optimal point, through excessive production or quenching of free radicals, results in sub‐optimal force production (reviewed in (Powers et al. 2011b)). Given the previously documented intensity‐dependent nature of free radical outflow from muscle (Bailey et al. 2003), we expected that the 5 km TT, a high intensity aerobic exercise, would elicit a significant increase in ROS which might contribute in the local development of fatigue and reduced performance. However, we may be the first study to report that acute treatment with the AOC did not improve, but in fact, attenuated cycling TT performance in young healthy males (Fig. 2). Our findings are somewhat in agreement with prior studies demonstrating no beneficial effects of vitamin C or vitamin E on endurance performance, though the majority of these studies chronically administered an antioxidant over time (Snider et al. 1992; Rokitzki et al. 1994; Bryant et al. 2003; Gaeini et al. 2006; Zoppi et al. 2006; Gomez‐Cabrera et al. 2008; Yfanti et al. 2010; Braakhuis 2012); which may (Gomez‐Cabrera et al. 2008; Morrison et al. 2015) or may not (Yfanti et al. 2010; Higashida et al. 2011) hinder the adaptation to exercise training. In fact, very few studies have investigated the potential acute effects on performance, but focused on whether antioxidants can reduce exercise associated ROS (Nakhostin‐Roohi et al. 2008; Teixeira et al. 2009). Though, some acute studies have demonstrated a positive impact of antioxidants (Medved et al. 2004), or a mixture of antioxidants (MacRae and Mefferd 2006; Abadi et al. 2013), on exercise performance. In contrast, using an AOC, containing vitamin C, E, and alpha‐lipoic acid did not improve, but rather hindered, endurance exercise performance.

Through the use of hyperoxic exposure we sought to determine the potential impact of elevating free radicals prior to exercise, on performance during a 5 km TT, a bout expected to raise ROS significantly, independent of the exposure to hyperoxia. Previously, hyperoxia has been used as a model to elevate free radicals (Freeman et al. 1982) and has been demonstrated to induce physiological dysfunction (Caruana and Marshall 2015). Indeed, prior studies have demonstrated that declines in performance are associated with elevations in free radicals/oxidative stress as a result of overtraining (Palazzetti et al. 2003, 2004). However, such studies have been correlative in nature and the stimulus of overtraining cannot simply be characterized by elevated free radicals only, as there are likely a myriad of issues such as psychological stress, immune changes, fatigue, etc. (MacKinnon 2000), thus making any direct implications of elevated free radicals on performance difficult. In the current study, using a stimulus known to increase free radicals and induce dysfunction (Freeman et al. 1982; Caruana and Marshall 2015), we did not find any impact of hyperoxic exposure on exercise performance. There are several possible explanations for this, such as the ROS associated with hyperoxia may have had a priming effect on the skeletal muscle (Reid et al. 1993), second, the dose of hyperoxia may have been insufficient, though previous work suggests otherwise (Mak et al. 2002; Gao et al. 2012), and finally our population of young healthy males, not subject to malnourishment, or overtraining, were likely capable of dealing with an acute free radical challenge. In summary, in the current model, exposure to hyperoxia does not appear to hinder nor improve cycling exercise performance.

### Antioxidants, hyperoxic exposure, and physiological responses

Few studies have focused on the acute response to antioxidant supplementation, or have often focused on the changes in blood markers (Gaeini et al. 2006; Nakhostin‐Roohi et al. 2008) which may (Medved et al. 2004) or may not (Rokitzki et al. 1994; Bryant et al. 2003; Zoppi et al. 2006) have any impact on, or relate to, exercise performance. In a previous study, MacRae and Mefferd (2006) found that an AOC containing essential vitamins and quercetin, a polyphenolic compound, improved cycling power output and performance during a 30 km TT without any significant impact on HR or *V*O_2_. Conversely, researchers who found no performance benefit of antioxidant supplementation also found no impact on HR, blood glucose, free fatty acids, lactate, or lactate threshold (Snider et al. 1992; Rokitzki et al. 1994). Collectively, these studies suggest that irrespective of performance outcome, antioxidant supplementation has no impact on the physiological response to exercise during a performance trial.

On the contrary, in this study we found that while HR and *V*O_2_ were unaffected, *V*_E_ was reduced, and blood lactate was elevated in the AOC trials as compared to placebo (Fig. 3). Importantly, the unchanged *V*O_2_ and HR, despite a lower power output, suggests a reduction in efficiency. Indeed, efficiency, as defined by the relationship between energy cost (*V*O_2_) and output (W), was found to lower with the AOC (Fig. 5) and is likely complementary to the reductions in TT performance. The findings from the current study are in contrast to those of MacRae and Mefferd (2006) highlighting the potential differences in antioxidant species. In the current study, the higher blood lactate associated with the AOC (Fig. 4) likely contributed to the reduced efficiency and impaired performance in the antioxidant trials.

As recently reviewed by Grassi et al. (2015), reduced efficiency and fatigue are not antithetical but highly related sharing similar mechanisms such as increased glycolytic flux and metabolite accumulation. Interestingly, in addition to the elevated blood lactate, the AOC also reduced ventilation, which could be contributed to the AOC‐induced reduction in power output. Although, given the unchanged *V*O_2_, a reduction in *V*_E_ actually suggests an improvement in the *V*_E_/*V*O_2_ (Fig. 3), and could be interpreted as an improved pulmonary efficiency. On the other hand, the reduction in *V*_E_ might be, at least in part, responsible for reduced acid‐base buffering, increased reliance on anaerobic glycolysis, and the elevated blood lactate in the AOC trials, which collectively contribute to reduced efficiency and performance. An alternative hypothesis is that adequate levels or free radicals are a necessary component of the hyperpnea associated with exercise, and excessive quenching of radicals with an AOC, might blunt this stimulus. To this end, using a rodent model, Delliaux et al. (2009), found that ROS (i.e., hydrogen peroxide) are key activators of metabo‐sensitive group IV afferents and suppression of these ROS with superoxide dismutase, blunted the ROS‐induced increase in afferent nerve activity. Thus, it is tempting to speculate that the AOC reduced free radicals, reducing group IV afferent activation thereby reducing ventilation. In agreement, work by Rossman et al. (2013)found that arterial infusion of ascorbate (Vitamin C), suppressed the ventilatory response to exercise in patients with COPD. Mechanistically, given the proposed contributory role of free radicals in mediating muscle function (Reid et al. 1993; Powers et al. 2011a), it is also possible that the AOC resulted in reductive stress and hindered respiratory muscle function and thus ventilation. Though, further work is needed to determine the potential impact of antioxidants on ventilation, particularly during exercise.

Previously, work in humans has demonstrated that normobaric hyperoxia enhances metaboreflex sensitivity during static exercise (Houssiere et al. 2006). However, we found no effect of a hyperoxic exposure on any of the physiological responses (i.e., HR, *V*O_2_, *V*_E_, blood lactate, etc.) to an exercise performance trial (Figs. 3 and 4). Though, it is important to note in the previous study by Houssiere et al. (2006) the hyperoxia was sustained during the exercise bout, unlike the current study which used a pre‐exposure, and likely explains the disparate results between the previous and current studies. In the current model, hyperoxic exposure prior to exercise had no effect on the physiological responses to exercise during a cycling performance trial.

### Antioxidants, hyperoxic exposure, and perception of fatigue

Previous studies investigating the impact of antioxidants on performance have either not reported perceptions of fatigue or are complicated by potential changes in power output. Indeed, prior work on antioxidants and fatigue has shown that through administration of NAC fatigue can be attenuated (Reid et al. 1994; Medved et al. 2004; McKenna et al. 2006), though not without invasive infusion or side effects. Similarly, Rossman and colleagues found that infusion of Vitamin C reduced both RPE and peripheral muscle fatigue in response to exercise, matched for time under placebo conditions, in patients with COPD. In contrast, in young healthy participants, we find that the oral AOC, containing vitamin C, E, and alpha‐lipoic acid, increased RPE, and left the post‐exercise visual analog scale for fatigue unchanged despite a lower power output (Fig. 6). However, it is important to highlight methodological differences in studies addressing antioxidants and fatigue; specifically time to fatigue or isotime exercise trials, are different approaches than the time trial used in the current study. Additionally, differences in antioxidant dose, composition, and population need to be considered.

Although there was no significant effect of condition or treatment on handgrip maximal voluntary contraction, it is interesting to note that in the normoxic trials we observed ~3% increase in post‐exercise handgrip MVC, likely as a result of post‐activation potentiation (PAP) (Xenofondos et al. 2015), which was not observed in the hyperoxic trials, suggesting PAP might be a redox sensitive phenomenon, but warrants further investigation. Moreover, since HG_MVC_ was unaffected by the AOC, suggests, potentially, that the impact of the AOC on RPE was more central, rather than peripheral, as the assumed reduction in free radicals could have altered afferent feedback, and/or central processing.

### Experimental considerations

As with other studies, this study is not without additional consideration. Namely, including assay of blood to evaluate free radicals/oxidative stress and antioxidant capacity would provide the supporting evidence for true representation of the redox balance and the potential perturbations elicited in the current protocol, as well as potential mechanistic insight into the current observations. However, previous studies have demonstrated the ability of antioxidants to reduce exercise associated ROS (Richardson et al. 2007; Nakhostin‐Roohi et al. 2008; Teixeira et al. 2009), but can yield vastly different effects on performance, strongly suggests, among other things, that functional consequences can differ dramatically from biomarkers. Finally, the possibility that the fat soluble vitamins used in the AOC (vitamin E) and fat and water soluble vitamin (alpha lipoic acid) might not have had enough time to wash out between visits cannot be excluded, though the counterbalanced design reduces, but does not eliminate this concern.

## Conclusion

The main findings of this study are that: exposure to hyperoxia prior to exercise failed to inhibit cycling performance; and the acute ingestion of an oral AOC does not improve, rather seems to hinder exercise performance, physiological functioning, or perceptions of fatigue in young healthy males. Thus, caution is warranted in athletes considering exogenous antioxidant supplementation for enhanced performance.

## Conflicts of Interest

The authors have no conflicts of interest to disclose.
